# Stable Single-Mode 795 nm Vertical-Cavity Surface-Emitting Laser for Quantum Sensing

**DOI:** 10.3390/ma17194872

**Published:** 2024-10-04

**Authors:** Yongli Wang, Yang Zhang, Chuanchuan Li, Jian Li, Xin Wei, Lianghui Chen

**Affiliations:** 1 Laboratory of Nano Optoelectronics, Institute of Semiconductors, Chinese Academy of Sciences, Beijing 100083, China; wyl17801127386@163.com (Y.W.); zhangyang@semi.ac.cn (Y.Z.); jalain@semi.ac.cn (J.L.); chenlh@semi.ac.cn (L.C.); 2 College of Materials Science and Optoelectronic Technology, University of Chinese Academy of Sciences, Beijing 100049, China

**Keywords:** VCSEL, 795 nm, single transverse mode, monolithic extended cavity

## Abstract

Vertical-cavity surface-emitting lasers (VCSELs) are essential for exhibiting single-transverse-mode output characteristics, which are critical for applications in quantum sensing, optical interconnection, and laser printing. In this study, we achieved stable single-transverse-mode lasing using extended-2λ-cavity with an oxide aperture diameter of 7.08 μm. The device demonstrated a high output power of 6.8 mW and a narrow linewidth of 49.8 MHz at room temperature. Additionally, it maintained stable single-mode emission at 794.8 nm and achieved a side-mode suppression ratio (SMSR) exceeding 40 dB within the temperature range of 25 °C~85 °C, thereby meeting the requirements of ^87^Rb atom quantum sensors. The fabricated device obtained high-power and narrow linewidth single-transverse-mode operation by a monolithic extended cavity without introducing additional processing procedures, which is expected to promote the commercial viability of VCSELs in quantum sensing.

## 1. Introduction

Quantum sensing technology has experienced rapid advancements in recent years, garnering significant interest from researchers across various fields. Notably, several types of quantum sensors, including atomic clocks, gyroscopes, and magnetometers, have emerged as focal points of investigation. The quantum sensor based on the ^87^Rb atom plays an irreplaceable role in the geophysical exploration and pharmaceutical industry due to its exceptional precision measurement capabilities. Vertical-cavity surface-emitting lasers (VCSELs) serve as critical light sources for quantum sensors owing to their attributes such as high spectral purity, circular output beams with low divergence, easy 2D integration, and potential for cost-effective mass production [1,2,3]. For ^87^Rb atom quantum sensors, a 795 nm VCSEL exhibiting single transverse mode, high power, and narrow linewidth is essential [4,5,6]. However, achieving single-mode operation in standard VCSELs, especially with high output power and narrow linewidth, poses a considerable challenge.

Traditionally, the single-mode operation has been facilitated by reducing the diameter of the oxide aperture to less than 4 μm. However, this approach causes high series resistance (typically exceeding 200 Ω for GaAs-based VCSELs), low output power (generally less than 3 mW), and a deterioration in reliability due to high current density and excessive current-induced self-heating [7]. To mitigate these limitations, various methods have been applied to achieve single-mode VCSELs with aperture diameters larger than 4 μm, including photonic crystal structures [8,9,10], surface relief [11,12,13,14], holey-structure integration [15], and elliptic mesas etching [16,17], which introduces selective gain or loss mechanisms in VCSELs to promote the dominance of the fundamental transverse mode. The largest oxide aperture diameter achieved for single-transverse-mode laser operation via these methods is 14 μm, with a maximum output power of 7 mW and a side-mode suppression ratio (SMSR) exceeding 40 dB. While these strategies have proven effective in facilitating single-mode operation [18], the increased complexity they introduce presents a challenge in terms of compatibility with mass-production processes.

Frank Schmidt’s research group successfully demonstrated the device operating in the fundamental transverse mode at oxide aperture diameters ~5 μm utilizing a double oxide layer configuration [19,20]. More recently, a single-mode VCSEL featuring an oxidation diameter of 6 μm along with an SMSR above 40 dB was realized by further displacing higher-order transverse modes from their gain region [21]. Reducing the overlap between the oxide layer and the standing wave field can decrease the number of active transverse modes present within the device structure [22,23,24,25]. Additionally, novel metal-mode-filtered designs have exhibited robust single-mode outputs even at an oxide aperture size of up to 8 μm [26]. A summary of these experimental findings is presented in Table 1.

In this study, we extend the inner cavity length to two-wavelength, which causes high diffraction losses for higher-order transverse modes, as higher-order modes with larger propagation angles suffer more diffraction losses than the fundamental mode. Ultimately, we demonstrate a stable single-transverse-mode operation VCSEL with a narrow line-width of 49.8 MHz and high power of 6.8 mW at 25 °C, all while remaining fully compatible with established oxide-confined VCSEL technologies without necessitating additional processing steps. Furthermore, we investigate how wavelength dependence varies with injection current across different temperatures and demonstrate stable performance within a wide operational range from 25 °C to 85 °C. Notably, at temperatures up to 85 °C, our device maintains stable during single-transverse-mode operation accompanied by an SMSR exceeding 40 dB.

## 2. Experimental Procedure

### 2.1. Crystal Growth

**Figure 1:**
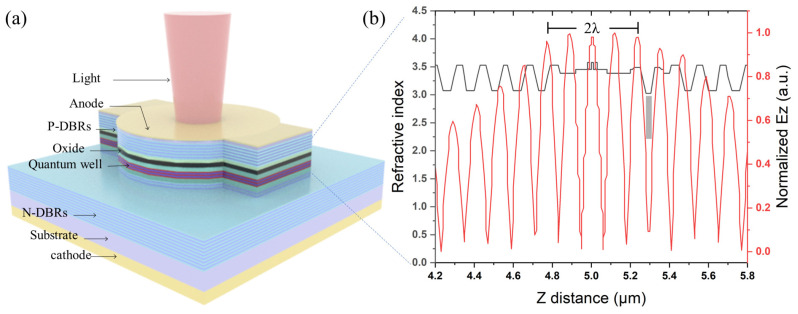
(**a**) Schematic of the epitaxial structure and fabrication of VCSEL; (**b**) refractive index and the longitudinal mode profiles in the growth direction for the VCSEL.

Figure 1a shows the schematic epitaxial structure of the investigated top-emitting VCSEL. The AXITRON AIX-200 metal–organic chemical vapor deposition (MOCVD) system was used for the structural epitaxy of VCSELs. Based on the GaAs (100) substrate, an n-doped distributed Bragg reflector (DBR) consisting of 40 pairs of Al_0.2_Ga_0.8_As/Al_0.9_Ga_0.1_As layers was grown. A three-layer compressive strain Al_0.12_Ga_0.765_In_0.115_As/Al_0.3_Ga_0.7_As quantum wells active region was sandwiched between two Al_0.3_Ga_0.7_As spacer layers. Additional Al_x_Ga_1-x_As layers were symmetrically added near the active region, and the Al composition needed to be carefully optimized. Ultimately, this forms a cavity with an optical thickness of 2λ (custom VCSELs typically have a cavity with an optical thickness of λ). A 30 nm oxide layer, formed by Al_0.98_Ga_0.02_As, was located between the low-Al content (Al_0.2_Ga_0.8_As) layer, which allows precise control of the aperture’s radius, thickness, and shape. Subsequently, 24 pairs of p-doped DBR were deposited. The interfaces within DBRs were graded in composition and doping concentration to minimize the free-carrier absorption and decrease electrical resistance. The refractive index along with standing wave field distribution near the active region is also depicted in Figure 1b.

### 2.2. Device Fabrication

**Figure 2:**
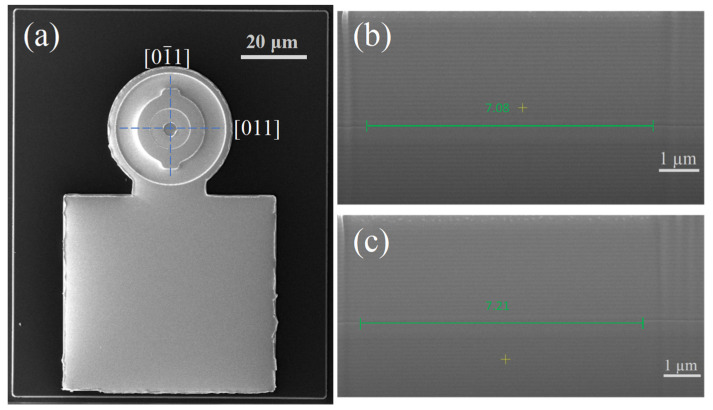
(**a**) SEM image of the top-emitting surface of the device; (**b**,**c**) are the cross-sectional SEM images of the device obtained by focused ion beam (FIB), with the oxidation diameter indicated along the [011] [011], respectively.

The as-grown material was then fabricated into VCSELs with different mesas using standard photolithography and dry etching techniques. Shallow mesa structures were etched to two or three pairs of n-DBR beneath the active region. Wet oxidation was then carried out in H_2_O and N_2_ environments, transforming the Al_0.98_Ga_0.02_As into a non-conductive insulation layer to form the aperture. The mesa was covered with a 400 nm SiO_2_ layer to isolate the Ti/Pt/Au metal contact. The GaAs substrate was thinned by about 100 μm and sputtered Ni/AuGe/Au. Subsequently, it was annealed at 480 °C for 190 s to improve the ohmic contact performance. The scanning electron microscopy (SEM) images of the VCSEL, presented in Figure 2, reveal oxidation diameters of 7.08 μm and 7.21 μm along two orthogonal directions.

### 2.3. Theoretical Simulation

**Figure 3:**
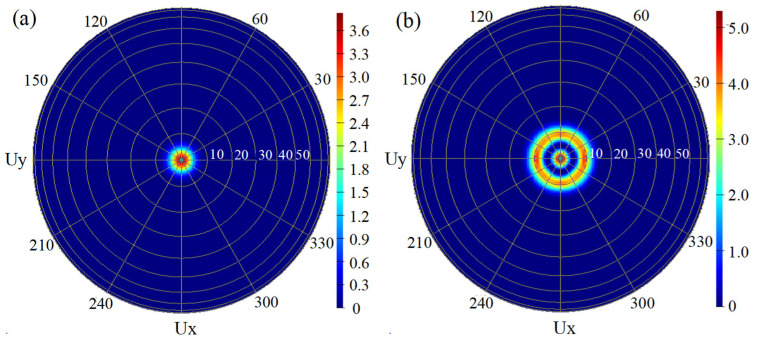
The far-field divergence angle distribution of (**a**) fundamental transverse modes, TEM00, and (**b**) higher-order transverse modes, TEM10.

The extended 2λ cavity, shown in Figure 1b, is beneficial for single-transverse-mode operation in a relatively large oxide aperture. The simulation results demonstrate that the far-field divergence angle of the fundamental transverse mode TEM00 is significantly lower than that of the higher-order transverse mode TEM10, as depicted in Figure 3. Consequently, as the cavity length increases, high-order transverse modes will suffer more diffraction losses due to their larger divergence angles than the fundamental transverse mode. The oxide layer is placed at the node location of the longitudinal optical mode, which is conducive to reducing the effective refractive index [27,28]. The effective refractive index can estimate the number of transverse modes within the VCSEL [29].

### 2.4. Measurements

The VCSEL chip for testing was mounted on a copper heatsink for temperature control and pumped under continuous wave conditions. The VCSEL was etched with a focused ion beam to form a cross-sectional structure, and the structure was characterized using a scanning electron microscope (HITACHI, Tokyo, Japan). The optical power was measured by a Thorlabs PM100D device with a S121C Si photodiode (Thorlabs, Newton, NJ, USA). The spectral characteristics of the device were measured using a YOKOGAWA AQ6370E spectral analyzer (YOKOGAW, Tokyo, Japan). A Fabry–Pérot (F-P) interferometer (Thorlabs, Newton, NJ, USA) with a free spectral range of 1.5 GHz was used to measure the linewidths of VCSELs.

## 3. Results and Discussion

### 3.1. Characteristic with Different Oxidation Diameter

**Figure 4:**
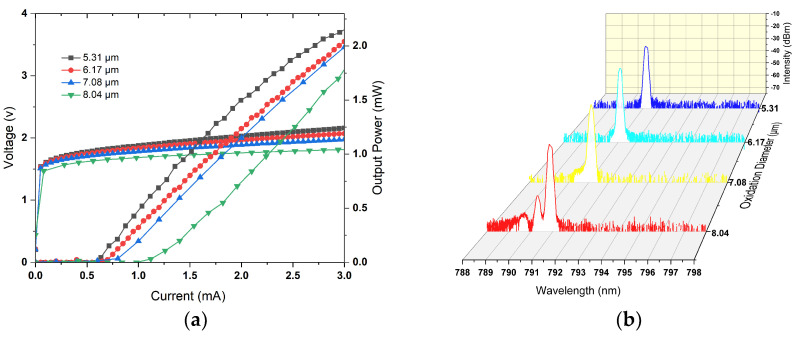
(**a**) P–I–V characteristics of VCSELs with different oxidation diameters; (**b**) the optical spectra of different oxidation diameters at an injection current of 2 mA.

Figure 4a presents the power–current–voltage (P–I–V) characteristic of VCSELs with different oxide aperture diameters. As the oxidation diameter increases, both the threshold current and resistance exhibit notable changes; specifically, the threshold current rises while resistance decreases significantly. This trend can be attributed to the expansion of the active region, which necessitates a higher current for adequate carrier density to initiate laser emission. Meanwhile, the samples with oxidation aperture ranging from 5.31 μm to 7.08 μm exhibit single-mode operation thanks to the 2λ cavity. However, as depicted in Figure 4b, when the oxidation aperture reaches 8.04 μm, the device transitions into higher-order modes of operation. This suggests that the optimal VCSELs support single-mode laser operation up to an oxide aperture diameter of 7.08 μm. The subsequent analysis will focus on evaluating the performance attributes of VCSELs featuring this specific aperture.

### 3.2. Characteristic with 7.08 μm Oxidation Aperture

#### 3.2.1. Power–Current–Voltage (P–I–V)

**Figure 5:**
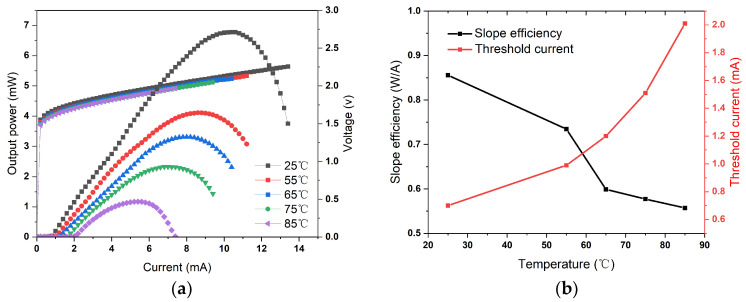
(**a**) P–I–V characteristics of the device under different temperatures; (**b**) the threshold current and slope efficiency as a function of work temperatures.

Figure 5a depicts the measured P–I–V characteristic of the sample with a 7.08 μm oxidation aperture at various temperatures. A typical rollover in the output power characteristics is observed as the driving current increases. The maximum output power decreases from 6.8 mW to 1.2 mW as the temperature rises from 25 °C to 85 °C. The high output power (6.8 mW) means that the laser can deliver a stronger signal, which helps improve the performance of quantum sensors such as atomic magnetometers. Concurrently, as the temperature increases, there is a significant rise in threshold current, and thermal rollover occurs earlier due to carrier thermal escape within the active region and an increase in losses [30]. The resistance of the device, determined by fitting the current–voltage curves, remains stable at 54 Ω across different temperatures and is significantly lower than that of GaAs-based VCSELs with smaller oxide diameters (~3 μm), which exhibit resistances around 200 Ω. This reduction in resistance can be attributed to the larger oxide diameter that enlarged the area of the active region. Lower resistance minimizes heat generation within the VCSEL, thereby improving its high-temperature operational characteristics. As depicted in Figure 5b, the slope efficiency of the device drops from 0.86 W/A to 0.56 W/A as the temperature increases because the effect of temperature on output power is more pronounced than that on the threshold current [31]. Additionally, Figure 5b presents threshold current as a function of operating temperature, revealing a minimum threshold current of 0.7 mA at 25 °C.

#### 3.2.2. Optical Spectra

**Figure 6:**
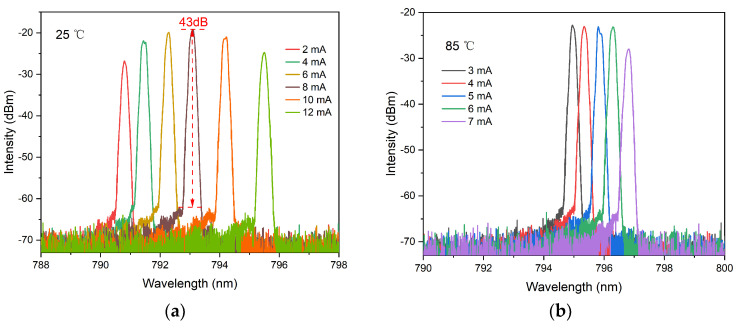
Current−dependent lasing spectra of the VCSEL under different temperatures. (**a**) 25 °C; (**b**) 85 °C.

Figure 6 displays the optical spectra characteristics of the VCSEL under different injection currents, utilizing a spectral analyzer with a measurement resolution of 0.02 nm. As injection current progresses from threshold levels toward thermal rollover, stable single-mode operation is maintained with high SMSR. A high SMSR means that the main laser mode has a higher intensity relative to the side mold, which can improve the signal-to-noise ratio of the quantum sensor and thus enhance measurement accuracy. The maximum SMSR recorded is 43 dB at 25 °C and drops slightly to about 40 dB at 85 °C. Stable single-transverse-mode output performance over a wide temperature range of 25 °C to 85 °C is essential for the long-term stability and reliability of quantum sensors. This consistent single-transverse-mode output can primarily be attributed to an extended cavity length that induces greater diffraction losses for higher-order modes due to their larger diffraction angles. Consequently, these higher-order modes are effectively suppressed across a wide range of injection currents. As the injection current increases, the lasing wavelength experiences a redshift due to the significant impact of elevated temperatures on the bandgap, as illustrated in Equation (1) [32]. At an operating temperature of 85 °C and an injection current of 2.8 mA, the lasing wavelength is measured at 794.8 nm, aligning with the designed specifications.

E_g_(T) = E_g_(0) − aT^2^/(T + b), (1)

where a = 6.2 × 10^−4^ eV/K, b = 280 K for GaAs.

**Figure 7:**
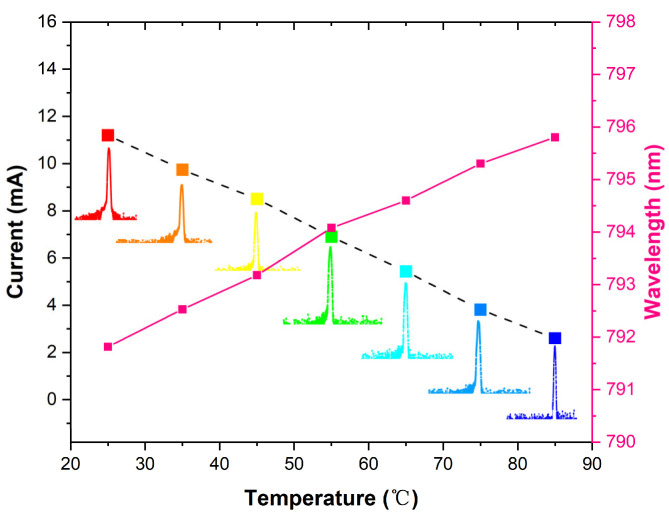
The black dashed line displays the injection current required for VCSEL operation at 794.8 nm under various temperatures, and the corresponding spectrum is also presented. The pink dotted solid line indicates the lasing wavelength of the VCSEL at different temperatures with an injected current of 5 mA.

The first fundamental optical transition corresponding to the D1 wavelength for ^87^Rb atom sensors is also noted at 794.8 nm [33], which defines the necessary wavelength for VCSEL operation. The emission wavelength is influenced by both injected currents and ambient temperatures. Figure 7 presents specific operating currents required to achieve a wavelength of 794.8 nm across varying temperatures.

The diverse emission spectra indicate that the device can stably operate in a single transverse mode over an extensive temperature range from 25 °C to 85 °C. Additionally, Figure 7 illustrates how optical output wavelengths depend on temperature, and a redshift in emitting wavelengths with increasing temperature has been observed. This phenomenon can be attributed to degradation in carrier confinement within quantum wells caused by accelerated internal self-heating effects at higher temperatures [8]. To mitigate thermal impacts, employing heatsinks with enhanced thermal conductivity may be beneficial. The fitting result indicates a redshift rate of approximately 0.067 nm/°C.

#### 3.2.3. Polarization

**Figure 8:**
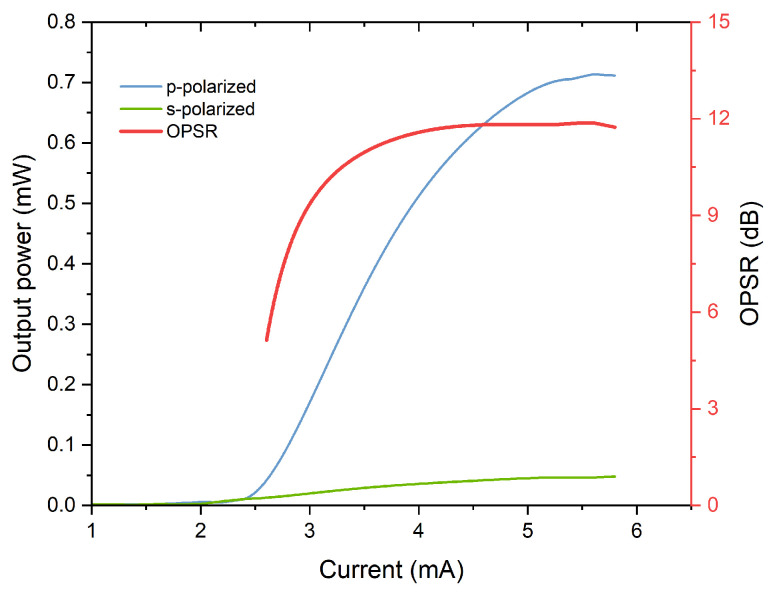
Polarization-resolved P-I characteristics of VCSEL at 85 °C.

Polarization characteristics are also important parameters for VCSELs in quantum sensing. Figure 8 displays polarization-resolved power–current (P-I) characteristic curves for VCSELs at an operational temperature of 85 °C. Notably, stable polarization operation is maintained up to an injection current of 6 mA. According to Equation (2), the maximum orthogonal polarization suppression ratio (OPSR) is approximately 11.8 dB. It should be noted that OPSR values for VCSELs typically fall short compared to those observed in edge-emitting lasers due to their symmetrical crystal planes and waveguide structures [34,35,36]. Reference [37] reports achieving a polarization suppression ratio as high as 21.5 dB through anisotropic grating structures. To enhance polarization characteristics and ensure stable polarization operation across all current levels, we plan to incorporate surface gratings in future designs.

OPSR = 10 × log(P_max_/P_min_), (2)

#### 3.2.4. Far-Field Patterns

**Figure 9:**
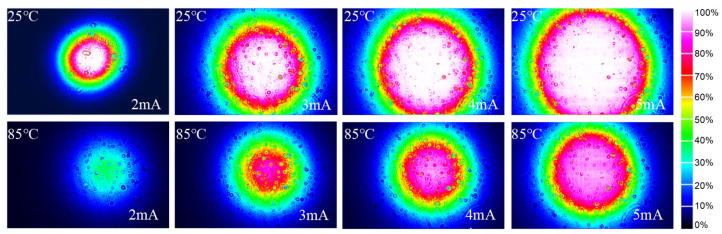
Far-field patterns of the 2λ cavity VCSEL at different currents and temperatures.

One of the advantages of VCSELs is their circular output beam profiles with small divergence angles. A beam profiler (Beamage 3.0) was positioned at a distance of 40 mm from the VCSEL to capture the far-field distribution under varying temperature and current conditions. As the injected current increases, both the central intensity in the far field and the beam diameter also increase due to a higher density of carriers in the active region, as illustrated in Figure 9. The observed reduction in far-field distribution of the beam diameter at high temperatures can be attributed to detuning effects between the cavity mode and active region gain. The near-circular profile distribution noted across all far fields indicates that the VCSEL operates within a single transverse mode, which is crucial for maintaining beam quality and coherence. The divergence angle, a critical parameter defining how laser beams spread during propagation, was calculated according to ref. [38], which provide a standardized method for measuring and reporting laser beam parameters. At an operating temperature of 85 °C with a bias current of 5 mA, we achieved a small divergence angle of 15.7° × 15.4°.

**Figure 10:**
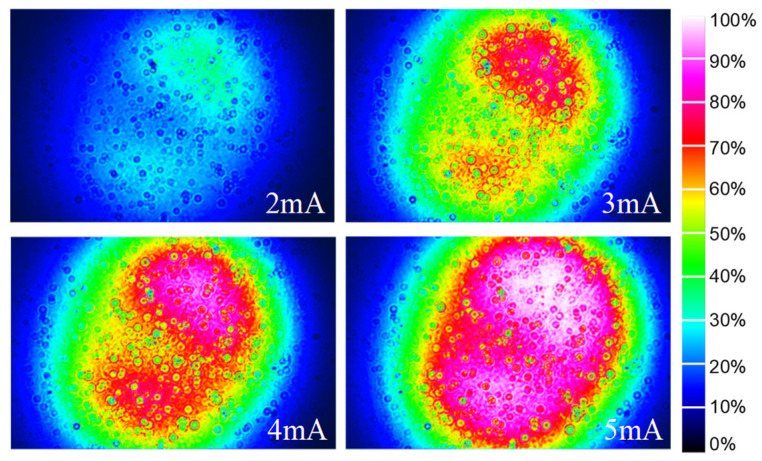
Far-field patterns of the VCSEL with λ cavity under different injection currents at 25 °C.

Simultaneously, we fabricated an identical VCSEL featuring a resonator cavity length of λ for comparative analysis. The far-field distribution patterns of this VCSEL under various injection currents at 25 °C are presented in Figure 10. Notably, high-order transverse modes emerge when employing an oxidation diameter of 4.44 μm. As the current rises, the intensity within these patterns rises, while light spots deviate from Gaussian distributions. As presented in the results, the single-transverse-mode output characteristics of VCSEL were optimized by extending the cavity length to 2λ.

#### 3.2.5. Linewidth

**Figure 11:**
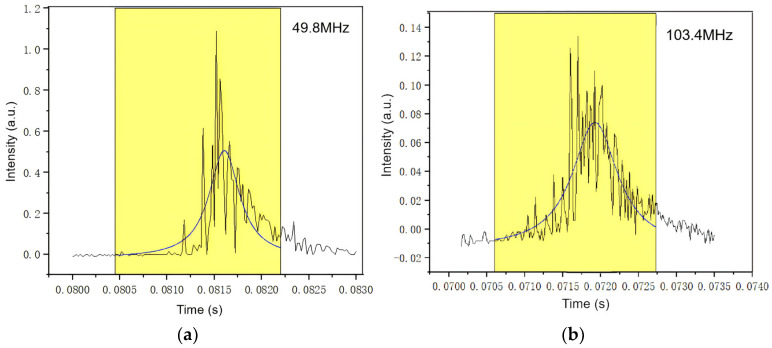
The linewidth of the VCSEL measured by FP interferometer at 2 mA. (**a**) The 2λ cavity; (**b**) the typical λ cavity.

By measuring the VCSEL at a bias current of 2 mA, we obtained its spectrum, which was subsequently fitted using Lorentz lines, as shown in Figure 11. The parameters of the F-P interferometer and controller were set to 1.5 GHz and 0.0118 s, respectively. Consequently, we calculated the Full Width at Half Maximum (FWHM) in MHz.

The mechanism behind the narrowing of the linewidth arises from the modification of the internal resonant cavity length of the VCSEL. By adding a light confinement layer to extend the cavity length to 2λ, we effectively increased the optical path length inside the cavity. This elongation enhances cavity precision, a measure of its ability to distinguish between different wavelengths, resulting in more selective filtering of cavity modes and leading to pronounced spectral discrimination alongside narrower linewidths [39]. As a result, the linewidth of our sample significantly decreased from 103.4 MHz to 49.8 MHz, indicating the influence of cavity length on the spectral characteristics of VCSEL. In quantum sensing, the linewidth of the laser needs to match the natural linewidth of the atom [33]. The reduction in linewidth can more accurately match the frequency of specific atomic transitions, thereby improving the signal-to-noise ratio and detection sensitivity of atomic transition signals. It is critical for applications demanding high spectral purity and stability, such as atomic clock systems and quantum magnetometers.

## 4. Conclusions and Future Outlook

In conclusion, we successfully fabricated a novel 795 nm VCSEL by extending the cavity length to 2λ and optimizing the location of the oxide layer. The sample demonstrates stable single-mode operation with a large aperture (7.08 μm) without any surface patterning, etching, or over-growth processes. It delivers an output power of 6.8 mW with a side-mode suppression ratio (SMSR) of 43 dB at room temperature. Excellent single-mode performance has been demonstrated across temperatures ranging from 25 °C to 85 °C. Furthermore, the sample achieves an impressively narrow linewidth of 49.8 MHz. We confirm the suitability of achieving large aperture, single-mode, narrow linewidth VCSELs by optimizing the epitaxial structure, which is conducive to promoting the commercial development of VCSELs in quantum sensing.

## Figures and Tables

**Table 1:** Comparison of the experimental results of the single-transverse-mode VCSEL.

Year	Method	Additional Steps	Oxidation Diameter (μm)	P_max_ (mW)	SMSR(dB)	Resistance (Ω)	Reference
1997	Adjust the oxide layer	no	3	2.25	35	-	[22]
2004	Holy structure	yes	~14	7	40	80	[15]
2007	Shallow surface relief	yes	6	6.3	30	84	[13]
2012	Surface relief with grating	yes	5	4.2	>20	-	[12]
2014	Double oxide layer	no	5		>20	90	[20]
2016	Patterned Dielectric	yes	3.75	3	≥25	-	[19]
2018	Photonic crystal	yes	-	3.7	>40	-	[10]
2018	Elliptic mesas	yes	6	0.55	30	-	[17]
2022	Adjust the oxide layer	no	6	4.85	41.68@80 °C	~50	[24]
2022	Adjust the oxide layer	no	6	4.85	41.68@80 °C	~50	[25]
2023	Metal-mode-filtered	no	8	-	-	-	[26]
2023	High-order transverse mode expansion	no	6	0.27	>40	300	[21]
**2024**	**Extended cavity**	**no**	**7.08**	**6.8**	**43**	**54**	**This work**

## Data Availability

The original contributions presented in the study are included in the article, further inquiries can be directed to the corresponding author.
